# IL-6 in Systemic Lupus Erythematosus: At the Intersection of Disease Activity and Cardiovascular Risk

**DOI:** 10.3390/jcm15135243

**Published:** 2026-07-04

**Authors:** Patricia Richter, Ciprian Rezus, Cristina Andreea Adam, Ioana Ruxandra Mihai, Alexandra Maria Burlui, Elena Rezus

**Affiliations:** 1 Department of Rheumatology and Rehabilitation, Grigore T. Popa University of Medicine and Pharmacy, 700115 Iasi, Romania; patricia.richter@umfiasi.ro (P.R.); mihai_ioana-ruxandra@d.umfiasi.ro (I.R.M.); maria-alexandra.burlui@umfiasi.ro (A.M.B.); elena.rezus@umfiasi.ro (E.R.); 2 I Rheumatology Clinic, Clinical Rehabilitation Hospital, 14 Pantelimon Halipa Street, 700661 Iasi, Romania; 3 Department of Internal Medicine, Grigore T. Popa University of Medicine and Pharmacy, 16 University Street, 700115 Iasi, Romania; 4 III Internal Medicine Clinic, “St. Spiridon” County Emergency Clinical Hospital, 1 Independence Boulevard, 700111 Iasi, Romania; 5 Department of Medical and Surgical Specialties I, II and III, Grigore T. Popa University of Medicine and Pharmacy, University Street No. 16, 700115 Iasi, Romania; adam.cristina93@gmail.com; 6 Cardiovascular Rehabilitation Clinic, Clinical Rehabilitation Hospital, Pantelimon Halipa Street No. 14, 700661 Iasi, Romania

**Keywords:** systemic lupus erythematosus, interleukin-6, cardiovascular risk

## Abstract

**Background/Objectives**: Interleukin-6 (IL-6) is a pro-inflammatory cytokine implicated in the pathogenesis of SLE. Beyond its role in disease activity, IL-6 has also been associated with increased cardiovascular risk, potentially promoting endothelial dysfunction, atherosclerosis, and thromboinflammation. Our study aimed to investigate the associations between IL-6 levels, disease manifestations, organ damage, cardiovascular comorbidities, and treatment regimens in a cohort of SLE patients. **Methods**: A total of 88 SLE patients were recruited from the Rheumatology Clinic of the Clinical Rehabilitation Hospital, Iași. Disease activity was assessed using the SLE Disease Activity Index (SLEDAI) and irreversible organ damage with the SLICC/ACR Damage Index. Serum IL-6 levels were measured by ELISA. Statistical analyses included Mann–Whitney U tests and Spearman correlation coefficients. **Results**: Among 88 SLE patients (89.8% female, mean age 51.9 ± 14.8 years), 68.2% presented irreversible organ damage, most frequently cardiovascular (26.1%). Regarding disease manifestations, IL-6 was non-significantly elevated in patients with arthritis, rash, and low complement levels. Serum concentrations also tended to increase with disease severity, being higher in severe compared to moderate activity, and in moderate versus mild activity. Significant associations were found between IL-6 and hypertension (*p* = 0.027), aortic atherosclerosis (*p* = 0.034), menopausal status (*p* = 0.015), and hypercholesterolemia (*p* = 0.034). No significant differences were observed across treatment subgroups. **Conclusions**: IL-6 showed limited correlation with SLE clinical activity but was significantly elevated in patients with selected cardiovascular comorbidities. These findings suggest a potential contribution of IL-6 to cardiovascular risk in SLE, warranting further investigation in larger cohorts.

## 1. Introduction

SLE is a chronic autoimmune disease with marked clinical heterogeneity, recurrent episodes of increased activity and potential involvement of multiple organs. Its manifestations range from mucocutaneous and articular involvement to severe renal, hematologic or neuropsychiatric complications. Although its etiology has not been fully elucidated, SLE results from a complex interaction between genetic susceptibility, hormonal influences, immune abnormalities and environmental triggers. A central pathogenic feature is impaired immune regulation, with autoantibody production, immune complex formation and defective clearance of apoptotic or necrotic cellular material. The persistence of these antigenic sources sustains abnormal immune activation, autophagy and cytokine release. Beyond classical organ damage, accelerated atherosclerosis and cardiovascular disease represent major contributors to morbidity and mortality in patients with SLE [1].

SLE is characterized by systemic autoimmune inflammation and is accompanied by an excess burden of premature atherosclerotic disease and cardiovascular events. Although therapeutic strategies have improved, cardiovascular involvement continues to substantially influence mortality in this population, even though many affected patients are young adult women. Evidence from large observational cohorts and systematic reviews shows that patients with SLE have a cardiovascular event risk approximately 2 to 5 times higher than that of the general population, with the relative excess being especially pronounced at younger ages [2].

Cardiovascular disorders represent a major category of comorbidities in patients with SLE. Available evidence indicates that individuals with SLE have a higher cardiovascular risk than age- and sex-matched patients with diabetes, and a risk more than twice that observed in the general population. Among cardiovascular events, myocardial infarction is frequently reported, with hazard ratios in SLE cohorts ranging from 2.6 to 5.1. Several factors associated with cardiovascular comorbidities in SLE have been described. These include demographic and physiological characteristics such as male sex, older age and postmenopausal status, lifestyle-related factors such as tobacco smoking, and conventional medical risk factors, including hypertension, hypercholesterolemia and diabetes mellitus [3].

Current guidelines recommend a thorough cardiovascular risk assessment in SLE, including:

- Conventional risk factors:
  - Age;
  - Hypertension;
  - Dyslipidemia;
  - Smoking;
  - Diabetes mellitus.
- Disease-specific factors:
  - SLE activity;
  - SLE duration;
  - Renal impairment;
  - The presence of antiphospholipid antibodies (aPLs);
  - Cumulative exposure to glucocorticoids [4,5,6,7].

The 2022 EULAR recommendations for cardiovascular risk management in rheumatic and musculoskeletal diseases, including SLE and antiphospholipid syndrome (APS), emphasize systematic evaluation and optimization of modifiable cardiovascular risk factors, together with the maintenance of low disease activity [8]. These measures include smoking cessation and regular physical activity as part of a healthy lifestyle, together with adequate pharmacological management of hypertension, hypercholesterolemia and diabetes mellitus [9].

Several medications used in SLE may adversely influence cardiovascular risk. Glucocorticoids are particularly relevant, as prolonged exposure or higher cumulative doses have been associated with increased cardiovascular event rates in SLE cohorts. This association may be partly explained by their broad immunosuppressive effects on innate and adaptive immune responses, together with adverse metabolic and hemodynamic changes, including increased blood pressure, dysglycemia and lipid abnormalities such as elevated cholesterol levels [3].

Osteoporosis and cardiovascular disease are frequent age-related conditions with shared risk factors and partially overlapping mechanisms. Low bone mineral density has been associated with increased cardiovascular morbidity and mortality, possibly reflecting common contributors such as aging, estrogen deficiency, vitamin D insufficiency, smoking and physical inactivity. Some therapies targeting bone or lipid metabolism may also influence this shared bone–vascular axis [10].

In SLE, the search for biomarkers of endothelial injury remains clinically relevant, particularly because endothelial dysfunction may precede overt vascular and organ damage. Interleukin-6 (IL-6) may be integrated into this framework as a proinflammatory cytokine with vascular relevance, since it can amplify endothelial activation, promote leukocyte recruitment and contribute to a cytokine milieu associated with cardiovascular involvement and disease progression in SLE [11].

The cytokine now known as IL-6 was initially identified under multiple designations. For instance, its ability to induce the differentiation of activated B cells into antibody-secreting plasma cells led to the designation “B-cell stimulatory factor 2” (BSF-2) [12]. Its effect on hepatic acute-phase protein synthesis prompted the name “hepatocyte-stimulating factor”, while its capacity to enhance the proliferation of hybridoma cells gave rise to the term “hybridoma/plasmacytoma growth factor”. Moreover, due to its antiviral activity, IL-6 was also referred to as interferon-β2 [13]. In 1986, the successful cloning of the BSF-2 cDNA by Hirano et al. [14] revealed that these various bioactive molecules, previously thought to be distinct, were in fact the same cytokine [13]. Consequently, the unified nomenclature ‘interleukin-6’ (IL-6) was adopted [15].

IL-6 is involved in several key processes: inflammation, immune response, metabolism and hematopoiesis. It also influences the endocrine and nervous systems, functioning as a cytokine with diverse biological effects [16,17]. IL-6 is considered a mediator of both innate and adaptive immunity, contributing to the integration of these two arms of the host immune response [18].

### 1.1. IL-6 Function in Immune Regulation

This integrative role becomes evident during inflammatory responses, where IL-6 is rapidly released by resident immune cells such as monocytes, neutrophils, and macrophages in response to tissue damage or infection. Toll-like receptor (TLR) stimulation, particularly by lipopolysaccharide (LPS), further induces IL-6 production in these myeloid cells. Additionally, activated myeloid cells release IL-1 and TNF-α, which promote substantial IL-6 synthesis from other cell types, such as endothelial cells and fibroblasts [18,19]. In SLE, IL-6, a crucial cytokine for the production of acute phase reactants and B cell maturation, is also increased and correlated with elevated autoantibodies and disease activity. Anti-IL-6 receptor monoclonal antibodies have demonstrated their success in both SLE patients and lupus mouse models [20]. Moreover, active clinical SLE is substantially predicted by the combination of circulating immune complexes and IL-6, superior to traditional biomarkers [21].

### 1.2. IL-6 and Cardiovascular Risk

SLE patients exhibit a notably increased cardiovascular risk, which is not adequately estimated by the traditional risk scores. In SLE, cardiovascular risk results from the cumulative effect of traditional and non-traditional determinants.

- Traditional cardiovascular risk factors include classical metabolic and lifestyle variables.
  - Smoking represents a modifiable behavioral determinant.
  - Hypertension is defined by persistent blood pressure values ≥ 140/90 mmHg.
  - Diabetes mellitus is identified by fasting glucose ≥ 126 mg/dL.
  - Dyslipidemia encompasses elevated total cholesterol ≥ 200 mg/dL, triglycerides ≥ 150 mg/dL, low HDL cholesterol < 40 mg/dL, and LDL cholesterol ≥ 150 mg/dL.
  - Metabolic syndrome integrates central obesity, glucose intolerance, hypertension, and lipid abnormalities. Overweight and obesity are defined by a body mass index ≥ 25 kg/m^2^.
  - A sedentary lifestyle reflects reduced physical activity and contributes to an adverse cardiometabolic profile.
- Non-traditional risk factors are related to disease-specific mechanisms and treatment exposure.
  - Persistent systemic inflammation is reflected by elevated inflammatory mediators such as TNF-α, IL-17, IFN-1, IL-6, and oxidized LDL.
  - aPLs include lupus anticoagulant, anticardiolipin, and anti-β2 glycoprotein I.
  - Hyperhomocysteinemia is defined by homocysteine levels > 15 μmol/L.
  - Increased C-reactive protein ≥ 1 mg/L indicates inflammatory activity.
  - Lupus nephritis represents renal involvement associated with an adverse vascular profile.
  - Treatment-related factors include prolonged or high-dose glucocorticoids ≥ 30 mg/day and methotrexate > 10–25 mg/week.

This dual framework highlights that cardiovascular risk in SLE extends beyond conventional cardiometabolic determinants and requires integration of disease-specific inflammatory, immunologic, renal, and therapeutic variables [22].

Classic scores, such as Framingham or ACC/AHA, tend to underestimate the cardiovascular risk in this population. Recent evidence supports the use of SLE-adapted tools, such as the SLECRISK algorithm, which integrates disease-specific variables (SLE duration, activity, renal function, and autoantibody profile), offering increased sensitivity in identifying moderate/high 10-year risk, especially in young women with severe forms of SLE [23].

The strategy for managing cardiovascular risk in SLE should include rigorous control of traditional risk factors: blood pressure (preferably through the use of renin–angiotensin system inhibitors, with additional benefits in SLE), lipid lowering (with early initiation of statins), glycemic control, and smoking cessation. Disease-specific measures include reducing exposure to glucocorticoids, achieving low disease activity, and administering hydroxychloroquine to reduce cardiovascular risk. Screening and treatment of antiphospholipid syndrome are essential, given its close association with arterial and venous events [4,6,7,24,25].

In certain categories of patients, non-invasive imaging methods, such as carotid ultrasound, can be helpful in detecting subclinical atherosclerosis, thus guiding the extent of cardiovascular risk reduction measures [5,26]. Nutritional interventions and lifestyle changes are recommended as adjuvant strategies for optimizing the cardiovascular profile [22].

The relationship between serum IL-6 and SLE, including immune dysregulation, disease activity, and clinical manifestations, was addressed in a previous article [27]. In the present study, the authors focus specifically on cardiovascular comorbidities in SLE, assessing their prevalence and their associations with traditional cardiovascular risk factors and SLE-related variables, including age at disease onset, disease duration, cumulative organ involvement, ongoing therapy, SLEDAI-2K, and SDI.

## 2. Materials and Methods

### 2.1. Patient Recruitment and Eligibility Assessment

We recruited 88 SLE individuals from the Rheumatology Clinic of the Clinical Rehabilitation Hospital, Iași, between July and November 2022. The cohort included both newly diagnosed and under-treated SLE patients. All the participants were required to sign an informed consent form.

The patients fulfilled the 1997 revised American College of Rheumatology (ACR) [28] or the 2012 Systemic Lupus International Collaborating Clinics (SLICC) classification criteria for SLE [29] or the 2019 (EULAR/ACR) classification criteria for SLE [30]. The inclusion of the 1997 ACR criteria was retained because the cohort included patients diagnosed over an extended period, including cases diagnosed before the publication and implementation of the more recent classification criteria.

All participants underwent screening for infectious diseases. Patients with active infections, other significant comorbidities, or active malignant tumors were excluded from the study.

The Ethics Committees of the Clinical Rehabilitation Hospital Iași and the “Grigore T. Popa” University of Medicine and Pharmacy, Iași approved this study.

### 2.2. Clinical Characteristics

Demographic and clinical parameters, including date of birth, gender, disease activity, organ involvement, comorbidities, and ongoing treatment, were retrieved from medical records.

We also assessed both the traditional cardiovascular risk factors including smoking, personal history of hypertension, hypercholesterolemia and/or hypertriglyceridemia, menopausal status, diabetes mellitus, and body mass index (BMI) and the SLE-specific parameters such as age at disease onset, disease duration, SLE Disease Activity Index (SLEDAI) score, and the Systemic Lupus International Collaborating Clinics Damage Index (SDI) for cumulative organ involvement.

Disease activity was evaluated during the clinical visit using the SLEDAI score, which reflects disease manifestations from the preceding 30 days, based on a composite score derived from 24 clinical and laboratory variables [31,32]. Disease activity was classified according to SLEDAI scores as follows: remission (SLEDAI = 0), mild activity (SLEDAI = 1–5), moderate activity (SLEDAI = 6–10), high activity (SLEDAI = 11–19), and very high activity (SLEDAI ≥ 20). This classification was used for disease activity stratification and should not be interpreted as formal DORIS remission, which requires additional clinical and therapeutic criteria.

In addition to disease activity monitoring, the assessment of SLE also requires evaluation of irreversible damage, permanent sequelae that impair the normal function of various organs and systems. The Systemic Lupus International Collaborating Clinics/American College of Rheumatology SLICC/ACR Damage Index is the first validated instrument developed to quantify cumulative organ damage in SLE. This index assigns a score of 1 or 2 points based on the severity of permanent, non-reversible structural changes attributable to the disease or its treatment. The scoring encompasses damage across multiple domains, including ocular, neurological, renal, pulmonary, cardiac, vascular, musculoskeletal, cutaneous, as well as endocrine (e.g., diabetes mellitus) and malignancy-related complications [33,34,35,36].

In evaluating comorbidities, particular attention was given to cardiovascular disease (CVD), classified into major subtypes (e.g., hypertension, ischemic heart disease, valvular heart disease, arrhythmias and conduction abnormalities, heart failure, aortic atherosclerosis, peripheral vascular disease, and venous disease).

Therapeutic regimens, including biologic agents, immunosuppressants, and corticosteroids, were also documented.

### 2.3. Quantification of Serum IL-6 Level

Blood samples were collected by standard venipuncture from SLE patients under aseptic conditions. After clotting at room temperature, samples were centrifuged at 3000 rpm for 10 min, and serum aliquots were stored at −80 °C until analysis.

Serum IL-6 concentrations were measured using a commercial human IL-6 ELISA kit based on monoclonal anti-human IL-6 antibodies, according to the manufacturer’s instructions (BioVendor, Brno, Czech Republic, catalog no. RD194015200R). Before analysis, serum samples were thawed, vortexed, and diluted 1:3 with dilution buffer. Standards, blanks, and diluted samples were added to antibody-precoated microplate wells and incubated according to the kit protocol. After sequential washing steps, biotinylated anti-IL-6 antibody and streptavidin-HRP conjugate were added, followed by substrate solution. The colorimetric reaction was stopped, and absorbance was measured at 450 nm using a microplate reader. IL-6 concentrations were calculated from a standard curve generated using recombinant IL-6 standards ranging from 1.25 to 80 pg/mL. Final concentrations were adjusted according to the dilution factor.

Routine hematological analyses were processed using standard procedures in our laboratory and interpreted according to reference ranges approved by the hospital.

### 2.4. Statistical Analysis

The normality of continuous variables was assessed using the Shapiro–Wilk test. Most variables showed a non-normal distribution, including IL-6, disease duration, age, SLEDAI score, triglycerides, glucose, CRP, and ESR, whereas BMI and total cholesterol did not significantly deviate from normality. Therefore, between-group comparisons were performed using the Mann–Whitney U test, and correlations between variables were assessed using Spearman’s rank correlation coefficient. Results are presented as mean ± standard deviation (SD) and median values. No formal correction for multiple comparisons was applied, as the analyses were exploratory and aimed to identify potential associations; therefore, *p*-values were interpreted in an exploratory manner. All statistical analyses were performed usingIBM SPSS Statistics, version 28.0; *p*-values < 0.05 were considered statistically significant.

## 3. Results

### 3.1. Descriptive Demographic and Disease-Related Data

Our study included 79 female patients (89.8%, mean age 51.9 ± 14.8 years) and 9 male patients (10.2%, mean age 44.4 ± 19.3 years) (Table 1).

Age at disease onset was most frequently reported after the age of 50, with 36.4% of patients having a late-onset form of SLE. Disease onset between 31 and 49 years accounted for 29.5% of cases, while 25% of patients experienced symptom onset between 19 and 30 years of age. Juvenile-onset SLE, defined as onset before the age of 18, was identified in 9.1% of the cohort.

The mean SLE duration was 10.38 ± 9.72 years, with values ranging from 0 to 37 years.

The prevalence of traditional cardiovascular risk factors was assessed in the study population. A positive family history of autoimmune rheumatic diseases was identified in 18.2% of cases, with a slightly higher frequency among females. Of the 79 female participants, 67.1% were post-menopausal. Additionally, 14.8% of patients were active smokers, with a higher prevalence in males.

According to the SLEDAI score, 21.6% of patients (n = 19) were in remission, 53.4% (n = 47) had mild activity, 21.6% (n = 19) had moderate activity, and 3.4% (n = 3) had high activity. No patients met the criteria for very high activity.

To further characterize active disease manifestations, a descriptive analysis was performed on the individual components of the SLEDAI score, assessing the frequency of each item within the cohort of 88 patients. The most frequent manifestations were serological abnormalities, with increased anti-dsDNA antibodies observed in 39 patients (44.3%) and low complement levels in 33 patients (37.5%). Among clinical features, rash was noted in 19 patients (21.59%) and alopecia in 12 patients (13.6%). Other manifestations, such as proteinuria (6.8%), arthritis (5.7%), myositis (3.4%), and mucosal ulcers (3.4%), were less common. Several features, including vasculitis, urinary casts, pericarditis, and fever, were absent in all patients.

Organ damage patterns were assessed by the SLICC/ACR Damage Index across the 88 patients with SLE. A score ≥ 1, indicating the presence of irreversible damage, was identified in 60 patients (68.2%). Cardiovascular involvement was the most frequent form of irreversible damage (26.1%), followed by renal (19.3%), central nervous system (13.6%), and musculoskeletal complications (11.4%).

Among the 88 patients evaluated, 12.5% had a confirmed diagnosis of diabetes mellitus. The mean body mass index (BMI) was 26.0 ± 4.91 (range: 15.56–38.00), with 60.2% of patients classified as overweight (BMI 25 ≥ kg/m^2^).

Regarding metabolic and biochemical abnormalities, hypercholesterolemia was present in 15.9% of patients, hypertriglyceridemia in 11.5%, and hyperglycemia in 14.8%. An inflammatory syndrome (high ESR and/or CRP) was documented in 43.2% of the cohort.

Descriptive analysis of treatment showed that 37.5% of patients received corticosteroids, while 6.8% were treated with NSAIDs. Hydroxychloroquine was the most commonly prescribed therapy, administered in 89.8% of patients. Among immunosuppressants, azathioprine was used in 34.1%, methotrexate in 8.0%, mycophenolate mofetil in 4.5%, cyclophosphamide in 3.4% and ciclosporin in 1.1%. Biologic therapy with belimumab was administered in 9.1% of patients.

### 3.2. IL-6 and SLE-Specific Parameters

Descriptive analyses and Mann–Whitney tests were performed to explore potential associations between IL-6 levels and SLEDAI manifestations. While IL-6 values tended to be higher in patients with arthritis and rash, these differences did not reach statistical significance (*p* = 0.082 and *p* = 0.132, respectively). Similarly, IL-6 concentrations were comparable between patients with and without alopecia (*p* = 0.601) or proteinuria (*p* = 0.843). No significant differences were found in relation to immunological parameters such as increased anti-dsDNA antibodies (*p* = 0.203) or low complement levels (*p* = 0.059). Overall, IL-6 did not show significant associations with these specific clinical features, although a non-significant trend toward higher levels in musculoskeletal, cutaneous, and immunological involvement was observed (Table 1). For the analyses presented in Table 2, patients were grouped according to the presence or absence of each individual clinical or laboratory manifestation, rather than according to a separate asymptomatic SLE subgroup.

**Figure 1:**
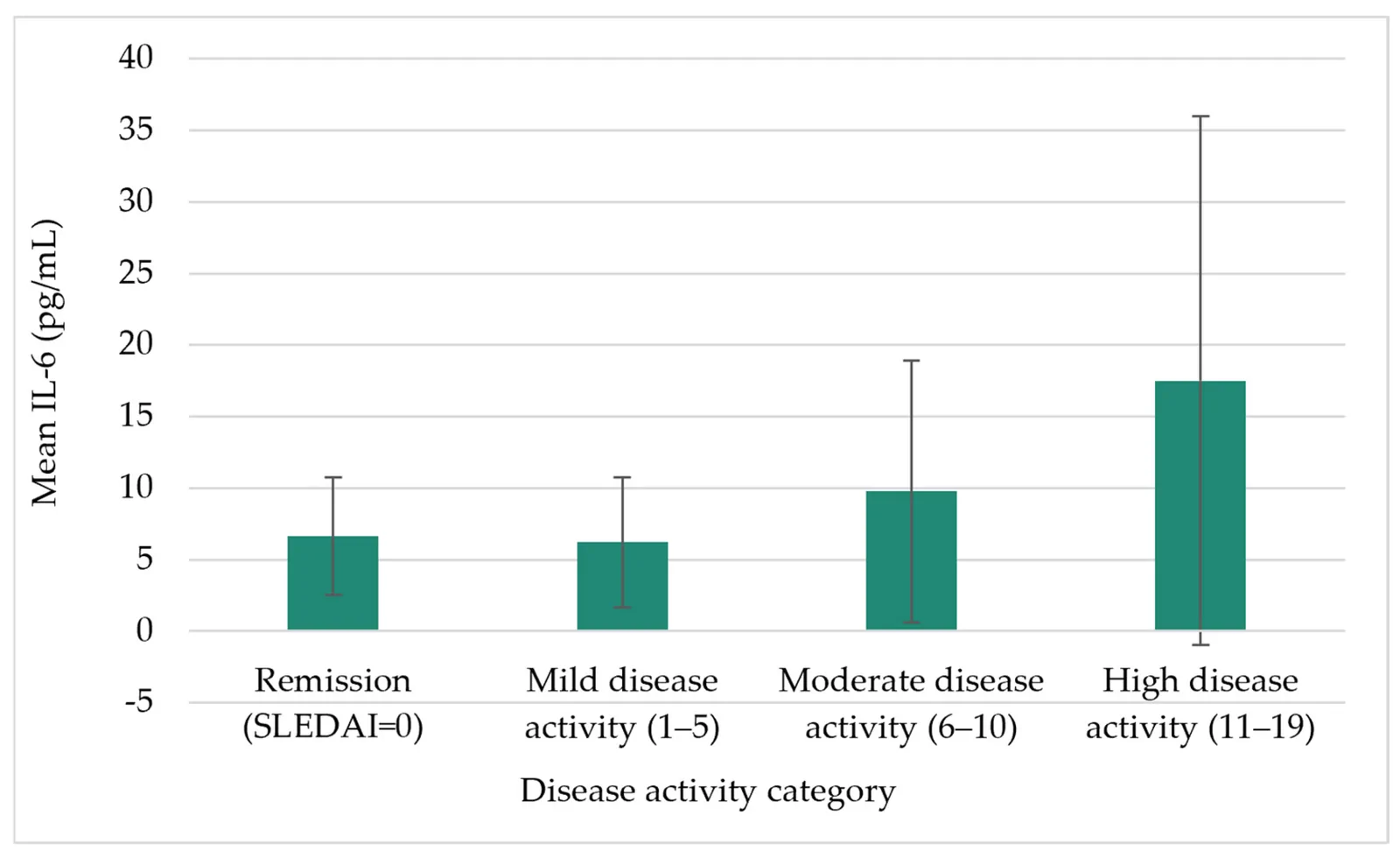
The bar chart illustrates mean IL-6 concentrations across SLE activity categories. IL-6 levels remained comparable between patients in remission and those with mild activity, but tended to increase in the moderate and high activity groups. However, these differences did not reach statistical significance.

Across disease activity categories, serum IL-6 levels showed numerically higher values in patients with moderate and high disease activity. Patients with SLEDAI = 0 had a mean IL-6 level of 6.63 ± 4.12 pg/mL, comparable to those with mild activity (SLEDAI 1–5), whose mean value was 6.22 ± 4.55 pg/mL. IL-6 levels were higher in patients with moderate activity (SLEDAI 6–10), reaching 9.77 ± 9.16 pg/mL, and were highest among those with high activity (SLEDAI 11–19), at 17.51 ± 18.50 pg/mL (Figure 1).

Subsequently, potential associations between IL-6 levels and different categories of SLE activity were explored. The Mann–Whitney U test was applied to compare IL-6 levels across remission, mild, moderate, and high activity groups. Although this upward trend suggested a possible association between IL-6 concentrations and disease activity, no statistically significant differences were identified between the groups (Table 3).

Regarding organ damage, IL-6 showed a positive and significant correlation with the numeric SLICC index when Spearman analysis was used (ρ = 0.28, *p* = 0.01). The Pearson correlation was weaker and not statistically significant (r = 0.17, *p* = 0.10). In univariate comparisons, IL-6 levels did not show statistically significant differences in patients with damage (SLICC/ACR Damage Index ≥ 1) versus those without damage in most organ domains.

A trend toward higher IL-6 values was observed in those with cardiovascular (*p* = 0.462), musculoskeletal (*p* = 0.202), and endocrine involvement (*p* = 0.103), though none of these reached statistical significance; an exception was the ocular domain (*p* = 0.045) (Table 4). For each variable, the comparator group consisted of patients from the same SLE cohort who did not present the respective manifestation.

### 3.3. IL-6 and Traditional Cardiovascular Risk Factors

IL-6 serum levels were analyzed in relation to smoking status, menopausal status, and family history of autoimmune diseases (Table 5).

No statistically significant differences were found between smokers and non-smokers (*p* = 0.114), nor between patients with or without a family history (*p* = 0.787).

IL-6 levels differed significantly based on menopausal status (*p* = 0.015, Mann–Whitney U test), with postmenopausal women showing higher concentrations (median: 5.3 pg/mL; mean rank: 44.38) compared to premenopausal women (median: 4.7 pg/mL; mean rank: 31.08).

The median serum IL-6 concentration in diabetic patients was 6.4 pg/mL, compared to 5 pg/mL in non-diabetic individuals. Despite this numerically higher median in the diabetic group, the difference did not reach statistical significance (Mann–Whitney U = 173.000, *p* = 0.227).

Osteoporosis was identified in 27.3% of patients (24/88), indicating a substantial prevalence of this condition in the study cohort. Serum IL-6 levels were significantly higher in patients with osteoporosis (*p* = 0.028).

No other significant correlations were observed with overweight status in the non-parametric analysis.

In the present analysis, both Pearson and Spearman correlation coefficients were employed to examine the relationship between serum IL-6 concentrations and selected metabolic parameters, including hyperglycemia, hypercholesterolemia, and hypertriglyceridemia.

Using Pearson’s correlation, no statistically significant associations were identified between IL-6 levels and any of the metabolic variables evaluated. The highest correlation coefficient was observed with hypertriglyceridemia (r = 0.107; *p* = 0.161), though this did not reach statistical significance.

In contrast, Spearman’s rank correlation revealed a modest yet statistically significant association between IL-6 and hypercholesterolemia (ρ = 0.196; *p* = 0.034), indicating that higher IL-6 levels may be modestly associated with the presence of elevated serum cholesterol. No other significant correlations were observed with hyperglycemia or hypertriglyceridemia in the non-parametric analysis.

These findings suggest a potential link between systemic inflammation, as reflected by IL-6 levels, and lipid metabolism, specifically hypercholesterolemia, in patients with SLE. However, the strength of the observed correlations was weak, and further studies are warranted to clarify these associations and their clinical relevance.

### 3.4. IL-6 Levels and Cardiovascular Comorbidities

**Figure 2:**
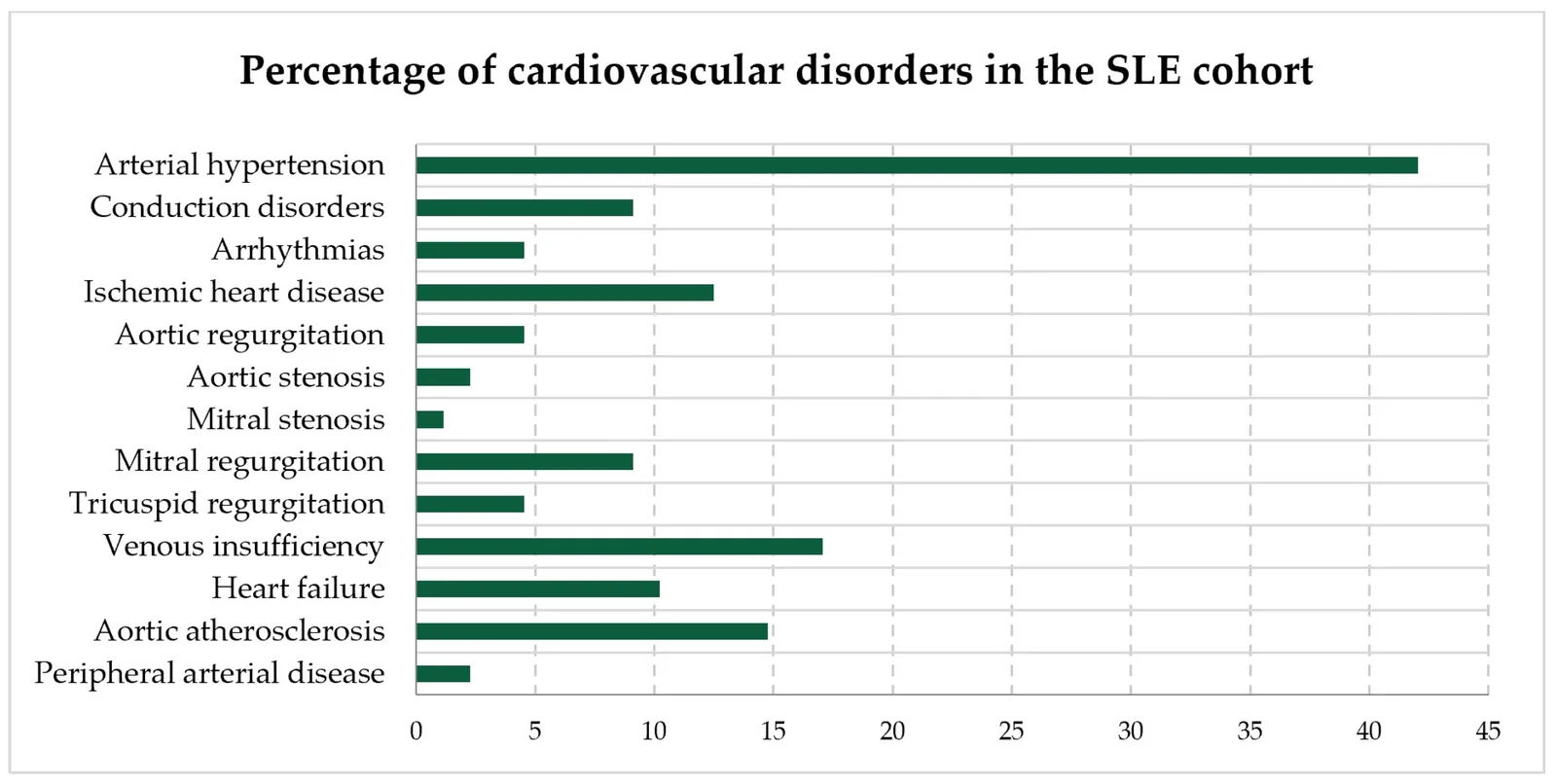
The horizontal bar chart illustrates the frequency of cardiovascular manifestations observed in the study cohort, with hypertension being the most prevalent condition.

In a cohort of 88 patients diagnosed with SLE, comorbid cardiovascular conditions were variably represented (Figure 2). We also analyzed serum IL-6 levels according to the presence or absence of various cardiovascular comorbidities. The most prevalent finding was arterial hypertension, reported in 42% of the patients. These individuals showed significantly higher IL-6 levels (8.8 ± 8.1 pg/mL) compared to those without hypertension (6.5 ± 5.4 pg/mL; *p* = 0.027).

Vascular pathology included aortic atherosclerosis in 14.8% of SLE patients and peripheral arterial disease in 2.3%. Chronic venous insufficiency was diagnosed in 17.0% of individuals. Additionally, a history of myocardial infarction was present in 6.8% of patients. In patients with aortic atherosclerosis, IL-6 concentrations were also significantly elevated, 11.1 ± 10.7 pg/mL vs. 6.8 ± 5.7 pg/mL in patients without this condition (*p* = 0.034). A markedly increased IL-6 mean was noted in the small subgroup with peripheral arterial disease, although the low number of cases limits meaningful statistical comparison. Individuals with a history of venous or arterial thromboembolic events (9.1%) had higher IL-6 levels (10.4 ± 13.2 pg/mL) compared to the rest of the cohort (7.2 ± 5.8 pg/mL), with a borderline statistical significance (*p* = 0.054).

Conduction system abnormalities were noted in 9.1%, while arrhythmias were documented in 4.5%. Ischemic heart disease was present in 12.5% of the cohort. Valvular abnormalities were also recorded with the following frequencies: aortic regurgitation in 4.5%, aortic stenosis in 2.3%, mitral regurgitation in 9.1%, mitral stenosis in 1.1%, and tricuspid regurgitation in 4.5%. Heart failure was identified in 10.2% of cases. For these comorbidities, no statistically significant differences in IL-6 levels were found.

### 3.5. IL-6 Levels and Antiphospholipid Antibodies

Antiphospholipid syndrome was associated in 19.3% (17/88) of the SLE patients.

IL-6 levels were examined in relation to antiphospholipid antibodies. The β2-Glycoprotein I (β2-GP-I) test showed a near-significant correlation with IL-6 (r = 0.281, *p* = 0.058). However, no statistically significant associations were identified between IL-6 and specific antiphospholipid antibody isotypes, including anti-β2-GPI IgA, IgG, and IgM, nor with anti-cardiolipin (aCL) IgG or IgM (Table 6).

### 3.6. IL-6 Levels and Treatment

Serum IL-6 levels did not differ significantly according to NSAID treatment (*p* > 0.05) or corticosteroid therapy (*p* = 0.578), based on the non-parametric analyses performed.

Correlation analysis showed an inverse association between prednisone dose and serum IL-6 levels. The Spearman correlation was statistically significant (ρ = −0.389, *p* = 0.025), suggesting that higher prednisone doses were associated with lower IL-6 levels. However, in the linear regression model, prednisone dose (mg/day) was not a significant predictor of serum IL-6 levels.

Serum IL-6 levels did not differ significantly according to the analyzed immunomodulatory or immunosuppressive treatments including hydroxychloroquine (Plaquenil) (*p* = 0.885) and the administered dose (200 vs. 400 mg/day; *p* = 0.442), as well as methotrexate (*p* = 0.493), azathioprine (*p* = 1.000), cyclophosphamide (*p* = 0.352 for current treatment; *p* = 0.350 for previous exposure), mycophenolate mofetil (*p* = 0.631), and belimumab (*p* = 0.095). These findings are limited by the small or unbalanced size of some treatment subgroups.

## 4. Discussion

Serological activity, particularly low complement and elevated anti-dsDNA antibodies, appeared to be the main contributor to disease activity, being present in 37.5% and 44.3% of patients, respectively. A non-significant trend toward higher IL-6 levels in these patients was also observed. The relatively low prevalence of renal (e.g., hematuria and proteinuria), hematologic (e.g., thrombocytopenia and leukopenia), and serosal (e.g., pleurisy and pericarditis) manifestations may reflect low disease activity or clinical remission at the time of evaluation. This pattern suggests a relatively low frequency of severe systemic or organ-threatening flares. Articular involvement, though common in SLE, was recorded in only 5.7% of our patients based on the SLEDAI score. IL-6 levels were higher in this group (*p* = 0.082), but the result was not significant, possibly due to a small number of patients. Among clinical manifestations, mucocutaneous features, including rash and alopecia, were the most frequent, while mucosal ulcers and myositis were rare. IL-6 tended to be higher in patients with rash (*p* = 0.132), but no significant associations were found with alopecia or proteinuria. Overall, the distribution of SLEDAI items in this study population highlights a profile dominated by serological and cutaneous activity, with a lower frequency of severe systemic involvement. While IL-6 did not demonstrate significant correlations with individual clinical or immunological manifestations, the non-significant trends toward higher values in patients with musculoskeletal, cutaneous, and serological features may reflect a possible contribution of IL-6 to the inflammatory milieu in a SLE cohort with predominantly mild to moderate disease activity.

In the literature, the frequency of these manifestations varied by demographic characteristics of the cohort and the disease activity. In large SLE cohorts, renal, joint, and cutaneous involvement are typically among the most common active manifestations. For instance, data from a lupus registry showed that active disease is often associated with serosal involvement, renal features (proteinuria or nephritis), and neurologic complications, including seizures [37]. In a cross-sectional study, treatment intensification in SLE was most commonly required in the presence of proteinuria, arthritis, and rash, while the utility of SLEDAI-2K as an indicator of disease activity was relatively limited [38]. Although the prevalence of individual SLEDAI items varies across SLE populations, renal, musculoskeletal, mucocutaneous, and hematologic manifestations are consistently among the most common [39]. Multiple reports examined the relation between IL-6 and the active clinical manifestations of SLE as measured with the SLEDAI score. Meta-analyses and cohort studies demonstrated significantly elevated in SLE patients compared to healthy controls, along with a positive correlation with SLEDAI scores, indicating higher IL-6 concentrations in patients with more active disease [40,41,42,43]. Regarding organ involvement, elevated serum IL-6 levels were associated with active lupus nephritis [43]. However, evidence on the association between serum IL-6 and specific clinical manifestations of SLE remains limited.

For evaluating irreversible damage in SLE, the SLICC/ACR Damage Index is considered a reliable measure for tracking organ damage over time [44]. Our findings reaffirm that cardiovascular and renal domains represent the predominant contributors to irreversible damage in SLE, consistent with previous literature emphasizing the cumulative burden of vascular and renal involvement over time. Although IL-6 values were elevated in subsets with specific organ damage, such as cardiovascular, endocrine, or musculoskeletal, these associations did not achieve statistical significance in our cohort. This may reflect limited sample sizes within subgroups or a multifactorial pathogenesis of chronic organ damage not fully captured by isolated cytokine levels. These findings highlight the predominance of cardiovascular and renal sequelae in long-term disease, underscoring the need for focused monitoring of these domains in SLE management. At the same time, the numerically increased IL-6 values in these categories may support further investigation into its potential contribution to long-term damage accrual.

While most studies have explored the relationship between IL-6 and disease activity, Mercader-Salvans et al. reported no significant association between circulating IL-6 levels and cumulative organ damage, as assessed by the SLICC/ACR Damage Index, suggesting that IL-6 may not correlate with long-term irreversible damage in SLE [1].

In this exploratory analysis, IL-6 levels were higher in SLE patients with selected cardiovascular comorbidities. In particular, patients with hypertension and aortic atherosclerosis showed higher serum IL-6 levels. However, these findings should be regarded as exploratory rather than confirmatory, given the largely unadjusted analytical approach. While elevated IL-6 was also noted in individuals with thromboembolic events and peripheral arterial disease, the statistical power was limited by small sample sizes. Nevertheless, these findings align with current evidence linking IL-6 to endothelial dysfunction, vascular remodeling, and thrombogenesis [45]. For other cardiovascular manifestations such as structural valvular defects (e.g., aortic or mitral insufficiency), conduction disorders, or ischemic cardiomyopathy, no statistically significant differences were observed. Overall, the data reinforce the association between IL-6 and vascular involvement in SLE, particularly in conditions like hypertension and atherosclerosis, where inflammatory cytokines may contribute to disease progression. Future studies on larger cohorts are warranted to clarify the pathogenic relevance of IL-6 across the full spectrum of cardiovascular complications in lupus.

Recent evidence showed that serum IL-6 levels were elevated in SLE and correlated with increased cardiovascular risk. Notably, IL-6 was independently associated with higher cardiovascular risk scores and unfavorable lipid parameters, specifically reduced HDL cholesterol and apolipoprotein A1 levels. However, these associations did not extend to global disease activity (SLEDAI) or SLICC/ACR DI after multivariate adjustment. These findings suggest that IL-6 may contribute to cardiovascular risk in SLE through mechanisms that are independent of overall disease activity or accrued damage [1].

Postmenopausal women showed higher IL-6 levels than premenopausal women. Estrogen (17β-estradiol) inhibits cytokine-stimulated IL-6 expression through estrogen receptor-mediated indirect mechanisms. The estrogen receptor can interact with transcription factors such as NF-IL6 and NF-κB and inhibit their DNA-binding activity, thereby reducing activation of the IL-6 promoter. This effect involves the proximal 225 bp sequence of the IL-6 promoter and does not require high-affinity binding of the estrogen receptor directly to IL-6 DNA. In addition, hormone therapy has been associated with reduced systemic inflammation, reflected by lower circulating cytokine levels, including IL-6 [46,47,48].

Hormonal status has been implicated in the regulation of IL-6, with particular attention given to estrogenic pathways. In postmenopausal women, reduced estrogen exposure has been proposed as a mechanism contributing to increased IL-6 production by peripheral blood mononuclear cells (PBMCs). Supporting this hypothesis, detectable IL-6 gene expression in unstimulated PBMCs has been associated with significantly lower serum estradiol levels. These observations suggest that postmenopausal estrogen deficiency may favor a more pronounced IL-6-related inflammatory profile [49].

Similar to our study, postmenopausal women showed significantly higher plasma IL-6 levels than premenopausal women [50]. In addition to reduced estrogen exposure, elevated FSH levels in menopause have been identified as a significant predictor of increased plasma IL-6, TNF-α, and IL-1β [50].

In our SLE cohort, no specific immunosuppressive or biologic agent was clearly associated with IL-6 levels. Previous studies have shown that corticosteroid therapy can suppress IL-6 production [40], while hydroxychloroquine (HCQ) treatment significantly decreased serum IL-6 concentrations after three months of administration [51,52]. Furthermore, belimumab therapy was associated with a sustained reduction in IL-6 levels starting from month 6 and continuing through month 24 of follow-up [53]. Mycophenolate mofetil (MMF) was also reported to significantly inhibit IL-6 mRNA expression [54].

This study has several limitations that should be taken into account when interpreting the results. First, the relatively small sample size, especially within patient subgroups, may have limited the statistical power to detect more subtle associations. This limitation could be addressed in future studies by recruiting larger cohorts and by performing predefined subgroup analyses with adequate statistical power. Additionally, the study was conducted in a single center, which may affect the generalizability of the findings to broader SLE populations with more diverse demographic profiles. Future multicenter studies including patients from different clinical settings would improve external validity and allow more robust assessment of interpopulation variability.

Second, the absence of a healthy control group prevents direct comparisons of IL-6 levels with those in the general population. This limitation could be overcome by including age- and sex-matched healthy controls, as well as disease-control groups when appropriate. Third, the cross-sectional design of the study precludes conclusions about causality. Given the dynamic nature of SLE—including disease flares, remission phases, and treatment-related fluctuations—a single time-point measurement of IL-6 may not allow for the evaluation of its longitudinal changes or predictive value for disease activity. Longitudinal studies with repeated IL-6 measurements and concomitant assessment of disease activity, treatment exposure, and cardiovascular outcomes would be needed to clarify temporal relationships and potential predictive value. Moreover, the majority of patients in our cohort were in remission or had mild disease activity, which may have led to an underestimation of the association between IL-6 levels and active disease states. Future cohorts should include a more balanced distribution of disease activity categories to allow more reliable comparisons across activity status. Finally, no vascular imaging, such as carotid Doppler ultrasound or intima-media thickness (IMT) assessment, was performed. These tools would have provided insights into the relationship between IL-6 and subclinical atherosclerosis, in the context of cardiovascular risk in SLE. Future protocols should integrate standardized vascular imaging, including carotid Doppler ultrasound, IMT assessment, or other validated markers of subclinical atherosclerosis, to better characterize cardiovascular involvement.

Prospective studies with larger, multicenter cohorts, inclusion of healthy controls, longitudinal follow-up, and integrated vascular assessment are warranted to validate and extend these findings.

## 5. Conclusions

In our SLE cohort, IL-6 levels were higher in patients with selected cardiovascular comorbidities, including hypertension and aortic atherosclerosis. These findings suggest possible links between IL-6 and cardiovascular comorbidity patterns in SLE, but they should be regarded as exploratory and hypothesis-generating rather than evidence of an independent association with cardiovascular risk. Further studies in larger, longitudinal cohorts are needed to elucidate the pathogenic and predictive value of IL-6 in SLE.

## Figures and Tables

**Table 1:** Descriptive demographic and disease-related characteristics of the SLE cohort.

Parameter	Value
Female patients	89.8%
Male patients	10.2%
Mean age in females	51.9 ± 14.8 years
Mean age in males	44.4 ± 19.3 years
Juvenile-onset SLE, <18 years	9.1%
Disease onset between 19 and 30 years	25%
Disease onset between 31 and 49 years	29.5%
Late-onset SLE, >50 years	36.4%
Disease duration	10.38 ± 9.72 years
Disease duration range	0–37 years
Positive family history of autoimmune rheumatic diseases	18.2%
Post-menopausal status among females	67.1%
Active smoking	14.8%
Diabetes mellitus	12.5%
BMI	26.0 ± 4.91 kg/m^2^
BMI range	15.56–38.00 kg/m^2^
Overweight status	60.2%
Hypercholesterolemia	15.9%
Hypertriglyceridemia	11.5%
Hyperglycemia	14.8%
Inflammatory syndrome	43.2%
SLEDAI-defined remission	21.6%
Mild disease activity	53.4%
Moderate disease activity	21.6%
High disease activity	3.4%
Very high disease activity	0%
Anti-dsDNA positivity/increase	44.3%
Low complement levels	37.5%
SDI ≥ 1	68.2%
Cardiovascular irreversible damage	26.1%
Renal irreversible damage	19.3%
Musculoskeletal irreversible damage	11.4%

Data are presented as percentages or mean ± SD, as appropriate. BMI, body mass index; SDI, Systemic Lupus International Collaborating Clinics/American College of Rheumatology Damage Index; SLE, systemic lupus erythematosus; SLEDAI, Systemic Lupus Erythematosus Disease Activity Index.

**Table 2:** Associations between IL-6 levels and individual SLEDAI-2K clinical and laboratory variables.

SLEDAI Manifestations	*p*-Value (Mann–Whitney U Test)
Vasculitis	-
Arthritis	0.082
Myositis	-
Urinary casts	-
Hematuria	-
Proteinuria/24 h	0.843
Pyuria	-
Rash	0.132
Alopecia	0.601
Mucosal ulcers	-
Pleurisy	-
Pericarditis	-
Low Complement	0.059
Increased DNA	0.203
Thrombocytopenia	0.483
Leukopenia < 3000/mm^3^	0.131
Fever	-

**Table 3:** Comparative analysis of IL-6 serum levels between patients with and without each SLE activity category.

Disease Activity	IL-6 (Mean ± SD) Present	IL-6 (Mean ± SD) Absent	*p*-Value (Mann–Whitney U Test)
Remission	6.63 ± 4.12	7.69 ± 7.30	0.601
Mild activity	6.22 ± 4.55	8.90 ± 8.40	0.131
Moderate activity	9.77 ± 9.16	6.83 ± 5.82	0.077
High activity	17.51 ± 18.50	7.11 ± 0.64	Not evaluated

**Table 4:** Distribution of irreversible organ damage in SLE patients and association with serum levels of IL-6.

Organ System Damage	*p*-Value (Mann–Whitney U Test)
Cardiovascular	0.462
Renal	0.132
Central Nervous System	0.243
Musculoskeletal	0.202
Pulmonary	0.635
Endocrine	0.103
Peripheral vascular involvement	0.226
Ocular	0.045
Cutaneous	0.739
Malignancy	0.899
Urogenital	0.417
Gastrointestinal	-

**Table 5:** Serum IL-6 levels according to traditional cardiovascular risk factors and metabolic variables.

Parameter	*p*-Value
Smoking status	*p* = 0.114
Family history of autoimmune diseases	*p* = 0.787
Menopausal status	*p* = 0.015
Diabetes mellitus	*p* = 0.227
Osteoporosis	*p* = 0.028
Overweight status	*p* = 0.387
Hyperglycemia	*p* = 0.362
Hypertriglyceridemia	*p* = 0.161
Hypercholesterolemia	*p* = 0.034

**Table 6:** Correlations between IL-6 levels and immunological markers.

Laboratory Parameters	Spearman’s Rank Correlation Coefficient (R)	*p*-Value
ACL screen	0.047	0.771
ACL IgA	−0.273	0.6
aCL IgM	−0.234	0.441
aCL IgG	0.134	0.648
B2GP screen	0.281	0.058
aβ2-GP I IgG	0.049	0.88
aβ2-GP I IgM	−0.237	0.51
aPL IgG	−0.015	0.91
aPL IgM	0.07	0.613

aCL = Anticardiolipin Antibodies; aβ2-GP I = Anti-β2-Glycoprotein I; aPL = Antiphospholipid Antibodies.

## Data Availability

The original contributions presented in this study are included in the article. Further inquiries can be directed to the corresponding authors.
